# Biospecimens, biobanking and global cancer research collaborations

**DOI:** 10.3332/ecancer.2014.454

**Published:** 2014-08-28

**Authors:** Camille Ragin, Jong Y Park

**Affiliations:** 1 Cancer Prevention and Control Programme, Fox Chase Cancer Centre, Temple University Health System, 333 Cottman Avenue, Philadelphia, PA 19111, USA; 2 African Caribbean Cancer Consortium (AC3); 3 Department of Cancer Epidemiology, Moffitt Cancer Centre, Tampa, FL 33612, USA; 4 Prostate Cancer Transatlantic Consortium (CaPTC)

**Keywords:** global cancer research, biospecimens, biobanking, biorepositories

## Abstract

The disparities in prostate cancer incidence and mortality continue to be a global public health problem. Efforts to address the prostate cancer disparity in black men have been met with a number of challenges, specifically in the accessibility to biospecimens in the context of global prostate cancer collaborations. During the International Educational Workshop at the Science of Global Prostate Cancer Disparities conference held 1–4 November 2012 in Nassau, the Bahamas, an overview of biobanking and biospecimen repositories, and materials transfer in global prostate cancer collaborations were discussed. The challenges faced by low-resource countries were identified, and potential solutions were recommended.

## Background and introduction

Biospecimen repositories have been established in many settings primarily to facilitate clinical and/or research purposes. The most common types of archived biospecimens include tissues such as blood, saliva, hair, nails, epithelia, soft tissues, urine, and other body fluids. These repositories play a significant role in cancer research by making biospecimens available for molecular and clinical characterisations in order to facilitate correlations with disease development or disease progression. Specifically, information may be generated that provides insight into disease mechanisms, identification of drug targets, and the development of screening tests, or identification of early detection biomarkers. Often times the purpose of the biospecimen repository dictates the type of biospecimens collected. For example, the longitudinal assessments of cancer risk might include normal tissues from persons without cancer over time, whereas collection of tumour and adjacent normal tissues from cancer patients might serve as resources for investigation of cancer aetiology, the outcomes or response to therapy.

The establishment of biospecimen repositories is not a new concept. One of the earliest was the US Armed Forces Institute of Pathology (AFIP). Since 1862, a DNA specimen repository for remains identification was maintained by the AFIP, but it was closed in September 2011. The AFIP’s functions are currently handled by the Joint Pathology Centre, which is the US federal government’s pathology reference centre, which now operates the National Pathology Tissue Repository [1, 2]. Other biospecimen repositories include the Cooperative Human Tissue Network, which is maintained by the US National Institutes of Health (NIH)/National Cancer Institute (NCI) and supports clinical research and clinical trials [3, 5]; the Prostate, Lung, Colorectal, and Ovarian (PLCO) Cancer Screening Trial, which maintains epidemiological data and biospecimens for approximately 155,000 participants [6]; and the newly established Prostate Cancer Biorepository Network (PCBN), which is funded by the US Department of Defense (DOD). The PCBN is a public resource that provides prostate cancer tissue and other biospecimens for prostate cancer researchers. The PCBN is a collaboration between the DOD, Johns Hopkins University, and The New York University School of Medicine [7]. Globally, epidemiologists in academic and clinical centres have also maintained population-based biospecimen collections that have supported longitudinal and individual research studies. An example is the European Perspective Investigation into Cancer and Nutrition (EPIC), a large cohort study including more than 520,000 participants that investigates the relationships between lifestyle, diet, nutritional status, and the environment on cancer incidence and other chronic diseases [8].

Cancer research has been accelerated through the utilisation of well-characterised and well-preserved biospecimens. For example, evaluations of 548 tumour specimens stored by the NCI Cooperative Breast Cancer Tissue Resource led to the development of the antibody trastuzumab, otherwise known as Herceptin [9], an effective therapy designed for a specific sub-population of breast cancer patients. A review of the global listing of biobanks, tissue banks, and biorepositories (www.specimencentral.com) [10] shows representation from several countries throughout Europe, Asia, North America, Australia, and the Middle East. While many advances have been made through the utilisation of carefully annotated biospecimen repositories, resources are still needed to address the global cancer disparities. In developing countries like Africa and the Caribbean Islands, where prostate cancer mortality rates are highest, well-established biospecimen resources equipped to facilitate global prostate cancer disparities research are limited.

### Evolution of biospecimens and current challenges

As new analytical technologies have developed over time, smaller samples can now be utilised. Thus, this helps to increase the variety of potential types of biospecimens that can be collected. However, this does not come without economic and technical challenges. There are issues surrounding space, cost, and storage. The existence of less racially/ethnically diverse biospecimen repositories also limits the applications of research findings, and many scientists have thus sought to pool specimen collections from multiple studies to increase sample size, statistical power, and diversity. This comes with many hazards, as there is a potential for introduction of bias due to variability in sample collection, processing, and storage, thus increasing the chance that some assay results may differ due to the varied conditions. Based on the above-mentioned challenges and potential biases, it is clear that there is a significant need for standardisation of biospecimen collection. Aside from the diversity in biospecimen collection, processing, and storage, further concerns exist for global collaborations in low-resource settings. The transfer of materials, ethical considerations, and legal constraints are also challenges.

## Methods and objective

The Science of Global Prostate Cancer Disparities in Black Men Conference was held 1–4 November 2012 in Nassau, the Bahamas. During the conference, one of the international educational workshops addressed biobanking and biospecimen materials transfer in global collaborations. The following includes the discussion points from this workshop. We highlight features of a biospecimen repository as well as best practices, with a specific focus on limitations and the potential solutions related to biospecimen collection, storage, and transportation in low-resource settings as part of global cancer research collaborations. The ethical and legal considerations in biobanking and biospecimen collection are also discussed.

## Results and discussion

### Technical and operational best practices

During the early 2000s the NCI established the Office of Biorepositories and Biospecimen Research (OBBR), and recommendations for best practices for biospecimen resources were first published in 2006. Subsequent updates yielded the current 2011 *NCI Best Practices for Biospecimen Resources*, which provides the principles for the development of biospecimen resources [11]. These recommendations promote best practices for the storage of quality specimens that yield quality data and are tailored to ensure adherence to legal and ethical requirements. The International Society for Biological and Environmental Repositories (ISBER) has also published a similar document, the 2012 *Best Practices for Repositories: Collection, Storage, Retrieval, and Distribution of Biological Materials for Research*, which provides effective strategies for biospecimen collection and management [12]. Both documents provide a set of adaptable standards to which repositories might adhere based on their purpose and setting. These include considerations for planning; required facilities and infrastructure; funding sources and other financial considerations; storage; the environment; ethical and legal issues; management of costs, records, and data; biosafety; training; biospecimen collection; quality control; as well as tracking retrieval and shipping. In the context of a low-resource setting, some of these recommendations may pose some challenges that could limit global cancer research collaborations.

To ensure scientific benefit of biospecimen collection in global cancer research collaborations, the management and operations of a biospecimen resource is critical for ensuring high-quality and utilisation. Careful attention must be paid to the organisational and infrastructure development, as well as operational efficiency. Irrespective of whether the biospecimen resource is small or elaborate, an organisational structure must first be developed and documented. The organisational structure of every biospecimen repository should include governance, management, resource support, and institutional support teams. The governance of biospecimen resources is critical for oversight and should comprise individuals or teams with appropriate expertise. Oversight committees, both external as well as institutional, should exist in order to ensure that the resource operations are conducted and managed with accountability and transparency. An oversight committee can play an important role in the planning and development of standardised, vetted, and approved internal policies and operational guidelines. Examples of important oversight committees include an advisory committee that provides scientific guidance and assist with strategic development of the resource. A biospecimen use committee, institutional review board, and/or materials transfer office would also play an important role in evaluating the scientific rationale and validity of a research study and the adherence to ethical and legal concerns, as well as enabling fair biospecimen allocation practices. The virtual non-existence of institutional review boards in many low-resource settings poses a direct threat to the organisational structure of a biospecimen repository and must be addressed. In the context of global collaborations, while these forms of oversight exist in high-resource partner countries, there must be also local oversight, emphasising the need to train and establish these committees within country to enable the smooth operations of global collaborations. Governance committees must also involve individuals with expertise in biospecimen management. Individuals must have first-hand knowledge of how a biospecimen resource should operate, and a lack of such expertise could pose a challenge since decisions and recommendations may not necessarily line up with the operational needs.

The management team plays a very important role in overseeing the day-to-day operations of the biospecimen repository as well as evaluating the quality of operations through development of procedural and regulatory standards. Managers must be familiar with best practices and ensure that their staff adheres to these practices and standard operational procedures. The responsibilities of a management team should include the selection and maintenance of equipment relevant to the operation of the biospecimen resource. A potential challenge in some low-resource settings, where power outages are common, is the assurance of continuous operation of instrumentation such as freezers and refrigerators. To maintain the integrity of the stored biospecimens, efforts should be undertaken to ensure that there is an adequate source of emergency power and/or generators. Other responsibilities of a management team include the purchasing and procurement of reagents and supplies from vendors. These responsibilities provide assurances of quality service and smooth workflow. Guidance in decision-making and the assessment of funding and overall service needs are therefore critical. In any biospecimen repository, adequate funding is necessary to sustain its activities. This may also be a challenge in low-resource settings where funding resources may be limited.

Vital to the adherence and application of operational procedures, the biospecimen resource support team of every biospecimen repository should include qualified and trained personnel, and education and training for them should be conducted regularly. For the most part, experts in pathology, epidemiology, clinical chemistry, biochemistry, molecular biology, and related disciplines involved in the establishment and maintenance of biospecimen repositories have provided on-the-job biospecimen science training to their laboratory personnel. Formal training programmes in biospecimen science will further impact the quality and standardisation of biospecimen collections and, ultimately, the quality of research. In some low-resource settings, access to trained personnel may pose a problem. This is especially because few specialised training programmes in biospecimen science exist and serve as models for training. Two training programmes endorsed by the ISBER include one sponsored by the University of British Columbia’s Office of Biobank Education and Research (OBER) and another sponsored by the Canadian Tumour Repository Network (CTRNet). Both of these training programmes provide online instruction [13]. Wider development, acceptance, and utilisation of biospecimen repository training programmes are highly recommended. In an effort to promote global cancer research collaborations, it is important that training be incorporated in all collaboration activities. When collaborations exist between investigators from high- and low-resource settings, some immediate solutions might involve scientific exchange programmes tailored to provide the appropriate training.

With every biospecimen resource, whether in a high- or low-resource setting, there must be institutional support teams that ensure smooth infrastructure and space planning. Infrastructure requirements will differ according to the setting and purpose of the biospecimen resource. Nevertheless, the existence of physical space for the processing and storage of biospecimens is the basic requirement. It is therefore critical, prior to the establishment of a biospecimen resource, that there is a full assessment of costs related to startup, operations, and maintenance. There must be adequate space allocated to biospecimen collection, processing, tracking, and shipping. Many of these needs cannot be addressed without strong institutional support. In some low-resource settings, strong institutional support for a biospecimen repository may prove to be a challenge as other priorities might take precedence when considering available funding, space, and maintenance costs.

### Biospecimen handling, processing and storage, and quality management

**Figure 1.**
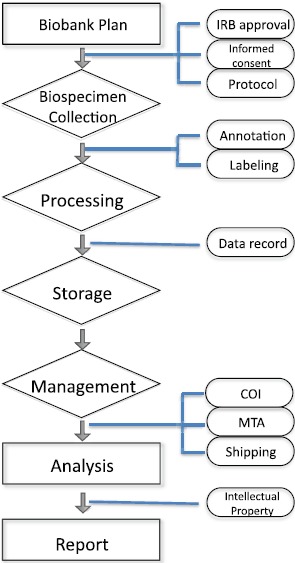
An overview of biobanking. Biobanks need solid planning and require IRB approvals, informed consent from participants, and a study protocol prior to the collection of biospecimens. These samples will be processed by annotation and labeling. Then, clinicopathological and epidemiological data will be recorded while the samples are stored appropriately. For analysis, samples will be released after checking conflict of interest (COI), and establishing a material transfer agreement (MTA) between institutes. Intellectual property issues need to be agreed upon before the results from analyses are reported.

Figure 1 provides an overview of the biobanking process. Every attempt should be made to minimise the effects of biospecimen handling on biospecimen integrity. Standardised protocols should be developed while considering factors that affect biospecimen quality pre, during, and post analysis. The establishment of global standards for biobanking and biospecimen collection is one potential solution that will impact the quality of research and address many challenges. These types of standards would address technical issues such as quality, consistency of collection, processing, and storage and will promote evidence-based biospecimen science. Pilot studies should be performed to validate new collection protocols, processing and storage protocols, as well as new equipment. The effects of multiple freeze–thaw cycles on blood analysis, special handling, processing, and storage of biospecimens for specific analytical platforms are a few examples of how standardised protocols help to ensure the quality of generated data.

Safety protocols should be in place to protect personnel, and inventory tracking systems should be in place to assure proper management and retrieval of biospecimens. A number of elaborate biospecimen management information systems exist that facilitate inventory management, utilisation, and tracking, but these may be cost prohibitive in low-resource settings. A well-organised ‘low-tech’ process that enables simple tracking and utilisation of biospecimens and is less expensive is more applicable in low-resource settings.

Quality management is another critical aspect of biospecimen repository science. Currently, quality control resources are available that can be utilised to assure that biospecimen repositories are operating optimally. Such resources include the International Society for Biological and Environmental Repositories’ Self-Assessment Tool (SAT) and the Biorepository Proficiency Testing programme, which is designed to evaluate how well repositories follow best practices. The SAT evaluates the accuracy of repositories’ quality control assays; compares proficiency ratings between repository laboratories worldwide, and performs staff proficiency and instrument performance evaluations. Similarly, the US College of American Pathologies accreditation and certification of Biorepositories is a new accreditation programme which requires the adaption of evidence-based standards and application of best practices. Utilisation of both examples are recommended for low-resource settings as this will strengthen the quality of biospecimen repositories, and thus ultimately promote and support stronger global research collaborations.

### Ethical, legal and policy best practices

In addition to the technical aspects of biospecimens and biobanking, there are several issues to be considered to build a global infrastructure for biospecimens and biobanking. A global biobanking system is expensive, and local support is essential. Ethical, legal, and policy issues are critical for the protection of participants, handling of biospecimens, and intellectual properties generated from research projects using biospecimens. The regulations for research using biospecimens in many countries are significantly different. Therefore, the researchers should be fully aware of regulations in collaborating countries. We will discuss several aspects related to ethical, legal, and policy issues.

All human research studies involving biospecimens are required to obtain an informed consent from participants, unless the research is exempt or granted a waiver under the international guidelines. A major challenge for informed consent is that all aspects of future studies cannot be anticipated when consent is obtained. The difficulty lies with the appropriateness of the informed consent document that does not allow flexibility in performing new assays as they develop or does not accommodate new methodologies, pathways, and targets that require special handling and preparation of samples. This challenge could limit the use of archived biospecimens. Therefore, careful thought must be given to the design and development of the informed consent document for biospecimen repositories. In addition, there are different challenges for informed consent in international studies due to limited resources and different cultural expectations. For example, in the United States, the signing of an informed consent form is mandatory for participating in research involving biospecimens. However, participants in certain areas, such as Caribbean and Middle Eastern countries, are often opposed to signing such documents.

The main role of an institutional review board (IRB) is to protect the rights, safety, and well-being of humans involved in research. In global research collaborations, one challenge is the literacy level of some participants and whether a verbal consent from them is good enough. Secondly, in some countries, wives are not supposed to make decisions on research participation without their husband’s approval. These issues need to be discussed with the IRBs in the United States and collaborating countries. Most institutions in the United States require an action according to their IRB policy when performing human subject research in other countries. For example, if equivalent boards do not exist in collaborating countries, investigators should try to obtain approval from local experts or leaders. The institutes in the United States may not approve protocols without documented approval from the collaborating countries.

In the United States, the privacy and confidentiality of patients’ personal data are governed by extensive regulations, such as the Health Insurance Portability and Accountability Act (HIPAA). However, in other countries, these regulations often do not exist or are difficult to enforce, if there are any, especially in some Caribbean Islands where the population size is small. Therefore, data protection and comparable regulations in collaborating countries are urgently needed. Evaluation of the status of confidentiality, communication with a local legal team, and establishment of the appropriate regulations for research in collaborating countries are critical for international research and successful global cancer research collaborations. Most molecular epidemiological studies require access to biospecimen and clinical data. The *NCI Best Practices for Biospecimen Resources* suggests three characteristics for guidelines for biospecimen distribution and data sharing [11]. The guidelines should be ‘clear, flexible, and amendable.’ However, some countries, such as China, govern strict regulations on human sample distribution to other countries.

Global research with biospecimens often generates patents that may have commercial values. Therefore, intellectual property and resource sharing is an important issue that must also be considered in global research collaborations. The *NCI Best Practices for Biospecimen Resources* suggested that researchers and institutions share research data and tools generated through the use of biospecimens in a timely manner [11]. This issue is extremely complex in the global setting because there is no consistent and comparable regulation across countries. Global cancer research collaborations generate knowledge that can be applied to detection, treatment, and prevention. Therefore, its economic impacts are enormous enough to be sensitive to intellectual property. Some progress has been made in understanding the interactions between intellectual property, regulatory and policy due to the international expansion of the industry, research and development (R&D), and trade. Comprehensive policy on this complex and critical area is in development, particularly in developing countries.

A conflict of interest (COI) involves the abuse of professional judgment for financial or non-financial interests. An example of a non-financial COI is that a handler of biospecimens is involved in research using the same biospecimen. However, sometimes these non-financial COIs are unavoidable, especially in small biospecimen repositories, which are often found in small countries. In this case, the COI should be disclosed clearly in public. Financial COIs may exist at the individual or institutional level. In 1995, the US Public Health Service enacted regulations regarding COIs. The key components of these regulations are: 1) an investigator must disclose any significant financial interest to the institution, and 2) an institution has the primary responsibility. It appears that COIs will always be there. However, most COIs, at least in academic settings, can be manageable if the high standard of ethical conduct of research is followed.

## Conclusions

A potential overall solution for successful global collaborations and smooth operations of biospecimen resources in low-resource settings is to emphasize training. The training of individuals in biospecimen science, ethics, and scientific leadership and management is imperative. This will build a pipeline of professionals who will be qualified to carry out the specific roles required for the smooth operation of a biospecimen repository. Funding resources designed to support biospecimen repositories in low-resource settings will also serve to strengthen global cancer collaborations. It is also imperative that there be local institutional buy-in knowing the importance of a biospecimen repository. Without strong institutional support and operational oversight, the existing challenges could impact the advancement of global cancer disparities research. Global best practice standards are also very important and should include ethical and legal issues such as the accessibility of specimens and data, criteria for information systems management, the construction of appropriate informed consent, and international coordination and sharing of biospecimens. Finally, the most important issue in global studies is to build mutual trust with researchers and institutes in collaborating countries.

## Conflicts of interest

The authors declare that they have no conflicts of interest.
